# PLAC8-Mediated Activation of NOX4 Signalling Restores Angiogenic Function of Endothelial Colony-Forming Cells in Experimental Hypoxia

**DOI:** 10.3390/cells12182220

**Published:** 2023-09-06

**Authors:** Shun Hay Pun, Karla M. O’Neill, Kevin S. Edgar, Eleanor K. Gill, Arya Moez, Hojjat Naderi-Meshkin, Sudhir B. Malla, Michelle B. Hookham, Mohammed Alsaggaf, Vinuthna Vani Madishetti, Bianca Botezatu, William King, Coy Brunssen, Henning Morawietz, Philip D. Dunne, Derek P. Brazil, Reinhold J. Medina, Chris J. Watson, David J. Grieve

**Affiliations:** 1 Wellcome-Wolfson Institute for Experimental Medicine, Queen’s University, Belfast BT9 7AE, UK; spun01@qub.ac.uk (S.H.P.); karla.oneill@qub.ac.uk (K.M.O.); k.edgar@qub.ac.uk (K.S.E.); eleanor.gill@dpag.ox.ac.uk (E.K.G.); amoez01@qub.ac.uk (A.M.); h.naderi-meshkin@qub.ac.uk (H.N.-M.); michelle.hookham@belfasttrust.hscni.net (M.B.H.); malsaggaf01@qub.ac.uk (M.A.); vmadishetti01@qub.ac.uk (V.V.M.); bbotezatu01@qub.ac.uk (B.B.); wking07@qub.ac.uk (W.K.); d.brazil@qub.ac.uk (D.P.B.); r.medina@qub.ac.uk (R.J.M.); chris.watson@qub.ac.uk (C.J.W.); 2 Patrick G Johnston Centre for Cancer Research, Queen’s University, Belfast BT9 7AE, UK; s.malla@qub.ac.uk (S.B.M.); p.dunne@qub.ac.uk (P.D.D.); 3 Division of Vascular Endothelium and Microcirculation, TUD Dresden University of Technology, 01307 Dresden, Germany; coy.brunssen@uniklinikum-dresden.de (C.B.); henning.morawietz@tu-dresden.de (H.M.)

**Keywords:** NOX4 NADPH oxidase, placenta-specific 8, endothelial colony-forming cells, hypoxia, angiogenesis, reactive oxygen species

## Abstract

Ischaemic cardiovascular disease is associated with tissue hypoxia as a significant determinant of angiogenic dysfunction and adverse remodelling. While cord blood-derived endothelial colony-forming cells (CB-ECFCs) hold clear therapeutic potential due to their enhanced angiogenic and proliferative capacity, their impaired functionality within the disease microenvironment represents a major barrier to clinical translation. The aim of this study was to define the specific contribution of NOX4 NADPH oxidase, which we previously reported as a key CB-ECFC regulator, to hypoxia-induced dysfunction and its potential as a therapeutic target. CB-ECFCs exposed to experimental hypoxia demonstrated downregulation of NOX4-mediated reactive oxygen species (ROS) signalling linked with a reduced tube formation, which was partially restored by NOX4 plasmid overexpression. siRNA knockdown of placenta-specific 8 (PLAC8), identified by microarray analysis as an upstream regulator of NOX4 in hypoxic versus normoxic CB-ECFCs, enhanced tube formation, NOX4 expression and hydrogen peroxide generation, and induced several key transcription factors associated with downstream Nrf2 signalling. Taken together, these findings indicated that activation of the PLAC8–NOX4 signalling axis improved CB-ECFC angiogenic functions in experimental hypoxia, highlighting this pathway as a potential target for protecting therapeutic cells against the ischaemic cardiovascular disease microenvironment.

## 1. Introduction

Tissue hypoxia is a characteristic feature of ischaemic cardiovascular disease caused by atherosclerosis and poor revascularisation [1,2], which is associated with endothelial dysfunction [3,4], while effective functional angiogenesis may restore blood flow to ischaemic tissues, including heart [5], retina [6] and limb [2]. Although current clinical management of ischaemic cardiovascular disease, which is largely achieved through pharmacological agents or percutaneous intervention, is generally effective in slowing progression, none of these therapies are capable of promoting revascularisation or tissue repair. In this regard, targeting of endothelial progenitor cells, which possess superior angiogenic and proliferative capacity, to ischaemic tissue has emerged as a promising approach for a specific vasoreparative treatment of cardiovascular disease [7]. Within this minor population of circulating immature endothelial cells, endothelial colony-forming cells (ECFCs) are well defined by vast clonogenic and functional capacity, high progenitor marker expression and low immunogenicity, underlying their significant therapeutic potential. Our previous studies indicated that ECFCs fully integrate with the microvascular endothelium in vitro in both a single monolayer or tube network and promote neovascularisation in vivo, whilst injection of ECFCs into models of human disease indicated capability to migrate to ischaemic sites and promote revascularisation [8,9,10]. Importantly, isolation of CD157^+^ vascular stem cells displaying typical properties of ECFCs from mouse tissues provided clear evidence of their existence in vivo [11]. Although ECFCs are found in the peripheral circulation, increased numbers of highly proliferative and easily expandible cells may be isolated from umbilical cord blood (CB-ECFCs) [9,12], which are also more angiogenic and less immunogenic than peripheral blood ECFCs [10,13]. Although CB-ECFCs show clear potential for allogeneic therapy, insufficient therapeutic efficacy and inhibition of normal angiogenic function by the ischaemic tissue microenvironment represent major barriers to clinical translation [14]. 

In this regard, we recently identified NOX4 NADPH oxidase, a major cardiovascular reactive oxygen species (ROS) source [15] and predominant isoform expressed in CB-ECFCs [16], as a key positive regulator of CB-ECFC function and signalling, which supports creation of a pro-angiogenic microenvironment in vivo and drives post-ischaemic revascularisation via long-term modulation of host signalling [16]. Specifically, we reported that intramuscular injection of CB-ECFCs with NOX4 plasmid overexpression (OE) into a murine hindlimb ischaemia model promoted blood flow recovery and tissue perfusion. These effects were linked with increased functional vasculature, eNOS activation, ROS generation and pro-angiogenic signalling, which were inhibited in CB-ECFCs with NOX4 knockdown (KD) [16]. However, precise mechanisms underlying NOX4-mediated CB-ECFC vasoreparative function within the ischaemic microenvironment remain poorly defined. Understanding these mechanisms is critical for overcoming current challenges with CB-ECFC tissue delivery towards clinical translation. In this study, we, therefore, focused on the specific impact of hypoxia as a central feature of ischaemic cardiovascular disease, which is known to impair ECFC function [17] and both regulates and is subject to regulation by NOX4-derived ROS as a key determinant of angiogenesis [18]. 

CB-ECFCs were first confirmed to display reduced angiogenic function in response to 48 h of hypoxia, associated with decreased NOX4 expression and downregulation of ROS-mediated signalling, which was partially restored by targeted NOX4 OE. Microarray analysis of CB-ECFCs exposed to hypoxia versus normoxia identified placenta-specific 8 (PLAC8) as a central upstream regulator of NOX4, which when targeted by siRNA KD, resulted in enhanced tube formation driven by NOX4 induction and downstream activation of hydrogen peroxide-mediated Nrf2 signalling. Taken together, these data indicated an important role for PLAC8/NOX4 signalling in protecting CB-ECFCs against hypoxia-induced angiogenic dysfunction, thereby highlighting this pathway as a potential target for increasing therapeutic efficacy within the ischaemic cardiovascular disease microenvironment.

## 2. Materials and Methods

### 2.1. CB-ECFC Culture and Hypoxia Exposure

CB-ECFCs were isolated from donor umbilical cords under local ethical approval and were immunophenotyped using flow cytometry prior to use of multiple clones for all experiments. Cells were cultured on type I collagen-coated flasks in endothelial growth medium (EGM2) supplemented with BulletKit (Lonza) and 12% foetal bovine serum (FBS) and cultured at 37 °C with 5% CO_2_ under normoxic (21% O_2_) or hypoxic (1% O_2_) conditions in a hypoxia chamber. For experiments, CB-ECFCs at passages 6 to 12 were plated for 24 h to allow for attachment prior to exposure to hypoxia (in pre-equilibrated media) or normoxia for 48 h and subsequent analysis. Some cells were pre-treated with sulforaphane (4 µmol/L) for 4 h to promote Nrf2 activation.

### 2.2. Cell Transfection

For NOX4 OE, up to 5 × 10^5^ cells were resuspended in 100 µL transfection solution and electroporated using the Amaxa^®^ system and Basic Nucleofector Kit for Primary Mammalian Endothelial Cells (Lonza, Basel, Switzerland) together with 1 µg of either pcDNA4/TO/myc-His empty vector (EV) or pcDNA4/TO/NOX4-myc-His A expression plasmid containing a full-length myc-tagged copy of NOX4. PLAC8 KD was achieved by transfection of 1 × 10^6^ cells with 20 nmol/L of SMARTpool siRNA (Dharmacon™, Lafayette, CO, USA) against PLAC8 or non-targeting scrambled (SCR) control using DharmaFECT1^®^ transfection reagent (Horizon Discovery, Waterbeach, UK) for 24 h. Transfected CB-ECFCs were maintained in EGM2 complete medium for at least 24 h until further experiments.

### 2.3. Reverse Transcription and Quantitative RT-PCR

Total RNA extraction and on-column DNase treatment for genomic DNA were carried out using the High Pure RNA isolation kit (Roche, Basel, Switzerland), according to the manufacturer’s instructions. RNA purity and concentration were assessed by NanoDrop and cDNA was synthesised from 1 µg RNA using the High-Capacity cDNA reverse transcription kit (Thermo Fisher Scientific, Waltham, MA, USA). Quantitative qRT-PCR was performed in 2 µL reactions for 45 cycles using fluorescent SYBR Green (Roche) with gene-specific primers (IDT; designed using UCSC genome browser) with the following sequences (5′-3′): *NOX4* (FW: ATGGTGGTGGTGCTATTCCT; RV: CTGAAACATGCAACGTCAGC), *NOS3* (FW: GCAGCCTCACTCCTGTTTTC; RV: GGTCTTCTTCCTGGTGATGC), *HMOX1* (FW: AAAGATTGCCCAGAAAGCCC; RV: CTGGATGTTGAGCAGGAACG), *PLAC8* (FW: GTTGTGACCCAACCTGGAGT; RV: TTCCACACAGACAGCATTCA), *AKT1* (FW: GCCCAACACCTTCATCATCC; RV: ACACCTCCATCTCTTCAGCC), *AKT3* (FW: CAGAGGCAAGAAGAGGAGAGA, RV: ACTTGCCTTCTCTCGAACCA) and *ACTB* (FW: GAAAATCTGGCACCACACCT; RV: TGGATAGCAACGTACATGGC). The fluorescent signals generated during PCR amplification were analysed on a LightCycler 480 system (Roche). Relative quantitative values were obtained using the ∆∆Ct method. 

### 2.4. Protein Extraction and Western Blotting 

Cells were lysed using ice-cold RIPA buffer containing phosphatase inhibitor (Thermo Fisher Scientific, Waltham, MA, USA) and protease inhibitor (Roche) prior to quantification using BCA assay (Thermo Fisher Scientific). Protein (20 µg) was resolved on a 10% SDS-polyacrylamide gel and electro-transferred onto a PVDF membrane (GE Healthcare, Chicago, IL, USA) before being blocked with 5% non-fat milk in Tris-buffered saline with 0.1% Tween-20 (TBS-T) for 1 h at room temperature. Membranes were incubated overnight at 4 °C with primary antibodies reactive to NOX4 (ab133303, Abcam, Cambridge, UK; 1:2000), NOX2 (ab129068, Abcam; 1:2000), HIF-1α (#610958, BD Biosciences; 1:500), eNOS (ab76198, Abcam; 1:1000), HO-1 (#PA5-77834, Invitrogen; 1:1000), Nrf2 (ab62352, Abcam; 1:500), PLAC8 (MA527363, Thermo Fisher Scientific; 1:1000), p-STAT3 (9134P, Cell Signaling Technology, Danvers, MA, USA; 1:1000), STAT3 (9132, Cell Signaling Technology; 1:1000), p-c-Jun (9261S, Cell Signaling Technology; 1:1000), c-Jun (9165S, Cell Signaling Technology; 1:1000), p-VEGFR2 (2478S, Cell Signaling Technology; 1:1000), VEGFR2 (67407-1-Ig, Proteintech, Rosemont, IL, USA; 1:5000) and β-actin (3700S, Cell Signaling Technology; 1:5000). After three washes with TBS-T, membranes were incubated for 1 h with their respective HRP-conjugated secondary antibodies at 1:2000 dilution in 5% non-fat milk/TBS-T. Three further TBS-T washes were carried out before protein bands were detected using Chemiluminescent HRP Substrate (Merck, Rahway, NJ, USA) and analysed using Image-J v1.54f (NIH). 

### 2.5. Cell Metabolic Activity

Cell metabolic activity was quantified by MTT assay. CB-ECFCs were plated on a 96-well plate at a density of 2500 cells/well and incubated with 12% FBS-containing EGM2 for 24 h to allow cell attachment prior to exposure to hypoxia or normoxia for 48 h. EGM2 was replaced with 0.5 mg/mL MTT reagent at relevant time points and CB-ECFCs were incubated at 37 °C for a further 4 h under maintained hypoxia or normoxia. The MTT reagent was then discarded, and formazan crystals formed by viable cells were solubilised in 100 µL DMSO at 37 °C for 30 min before absorbance was measured at 570 nm on a FLUOstar Omega microplate reader (BMG Labtech) and normalised to percentage of the normoxia control.

### 2.6. Tubulogenesis Assay

CB-ECFCs were pelleted and mixed with growth factor-reduced Matrigel (Corning Inc., Corning, NY, USA) and complete media at a ratio of 60:40. A 40 µL Matrigel blob containing 7 × 10^4^ cells was spotted per well in a 24-well plate and incubated for 30 min at 37 °C to polymerise prior to addition of complete media and incubation for 48 h under either hypoxia or normoxia. The Matrigel blobs were then rinsed with PBS and incubated with 2 µg/mL cell-permeant Calcein-AM (Thermo Fisher Scientific) in Hank’s balanced salt solution (HBSS) for 1 h at 37 °C for staining before being replaced with fresh HBSS. Confocal imaging of tubes at 10× magnification (Nikon Eclipse TE2000-U) was conducted prior to quantification of tube area in three blobs analysing five images/blob using Image-J software (NIH). 

### 2.7. Immunocytochemistry

CB-ECFCs were seeded on a collagen-coated glass slide and incubated at 37 °C overnight to allow for attachment. Cells were then treated with 4 µmol/L sulforaphane (SFN) or DMSO vehicle control DMSO (4 ppm) for 4 h before being fixed with 4% paraformaldehyde in PBS for 10 min. After three washes with PBS, cells were permeabilised and blocked using PBS containing 0.1% Triton X-100 and 1% goat serum for 1 h at room temperature. Cells were then incubated with Nrf2 antibody (ab129068, Abcam; 1:250) in PBS containing 1% goat serum overnight at 4 °C. After a further three washes with PBS, cells were incubated with Alexa Fluor-594-conjugated secondary antibody (A11012, Invitrogen; 1:500) in PBS with 1% goat serum for 1 h at room temperature. After a final three washes with PBS, the cells were incubated with 1 µg/mL DAPI (D1306, Thermo Fisher Scientific) for 10 min before being mounted with Vectashield (H1000, VectorLabs) and imaged under a DMi8 fluorescence microscope (Leica, Wetzlar, Germany).

### 2.8. Cellular ROS Generation

Superoxide production was determined by staining CB-ECFCs with dihydroethidium (DHE; Sigma, St. Louis, MO, USA), which is specifically oxidised to ethidium by superoxide, while hydrogen peroxide production was quantified using a commercially available assay kit (ab138874, Abcam), according to the manufacturer’s instructions. For superoxide assay, cells were seeded on a 24-well plate at a density of 2 × 10^4^ per well. For hydrogen peroxide assay, the cells were plated on a 96-well plate at a density of 5 × 10^3^ per well, followed by culture under either hypoxia or normoxia for 48 h. Cells were then washed twice with a PBS and incubated with 10 µmol/L DHE in HBSS or AbGreen hydrogen peroxide indicator solution for 1 h at 37 °C. The staining solutions were replaced with HBSS for fluorescent imaging (Leica DMi8) with quantification in three wells analysing three images/well using LAS X v3.7.4 (Leica).

### 2.9. Microarray Analysis

RNA was extracted from CB-ECFCs and reverse transcribed to cDNA for microarray analysis (Cambridge Genomic Services) using an Illumina HumanHT-12 v4 BeadChip array system. Raw gene array data were normalised and imported into Partek Genomics Suite (PGS) software and R/RStudio (version 4.2.1; limma, ggplot2) for statistical analysis, including visualisation using principal component analysis, volcano plot and differential gene expression (both upregulated and downregulated) using analysis of variance (ANOVA via PGS) or limma R package. A gene list was generated and uploaded to Ingenuity Pathway Analysis (IPA, Qiagen, Hilden, Germany) for network analysis to identify NOX4-associated pathways based on a fold change of ± 1.5 and *p* < 0.05.

### 2.10. Human Phospho-Kinase Proteome Profiler Array

Targeted analysis for protein phosphorylation was performed in CB-ECFC lysate using a human phospho-kinase proteome profiler array (ARY003C; R&D Systems, Minneapolis, MN, USA). Equal concentrations of protein lysate derived from three CB-ECFC clones in triplicate were pooled (300 µg in total) and incubated with antibody pre-loaded membranes, according to the manufacturer’s instructions. The pixel density of each spot was quantified using HLImage++ vPCM.22.0.1.b (Western Vision Software) with duplicate spots for each target averaged and normalised to the three reference spots on each membrane. Relative changes in protein phosphorylation were presented as a heat map, generated using Microsoft Excel for Windows 365, with increased levels indicated in green and decreased levels shown in red.

### 2.11. Statistical Analysis

Data were expressed as mean + the standard error of the mean (SEM). An unpaired Student’s *t*-test was used to assess statistical significance between two groups, whereas a comparison between multiple groups was performed using a one-way ANOVA with Bonferroni post-hoc testing using GraphPad Prism v10.0.2. In all cases, *p* < 0.05 was taken to indicate statistical significance. All data were checked for statistically significant outliers using Grubbs test.

## 3. Results

### 3.1. Hypoxia Promotes CB-ECFC Angiogenic Dysfunction and Reduced ROS Production

**Figure 1:**
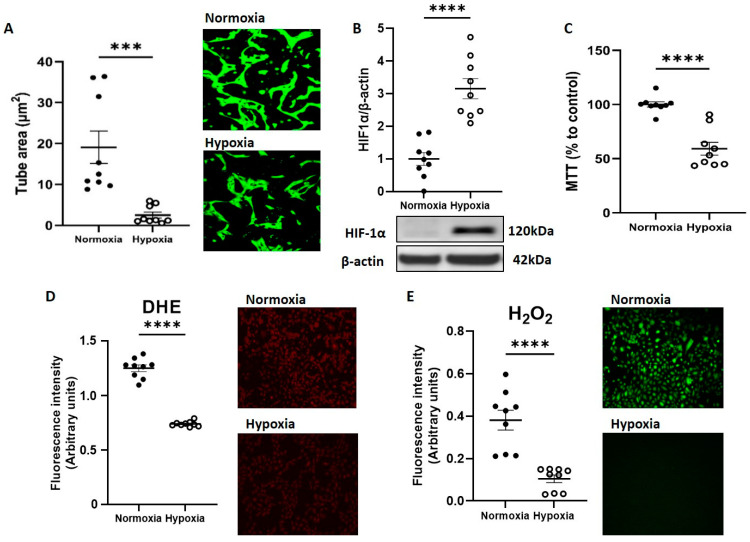
**CB-ECFC angiogenic function and ROS generation were reduced by hypoxia exposure.** CB-ECFCs were cultured under hypoxia or normoxia for 48 h prior to endpoint assessment. (**A**) Matrigel tubulogenesis assay with quantification of the tube area. (**B**) HIF-1α protein expression determined by Western blot and normalised to β-actin. (**C**) Cell metabolic activity assessed using MTT assay. (**D**) Superoxide and (**E**) hydrogen peroxide production measured by DHE staining and commercially available assay kit, respectively. Representative cell images and Western blots are shown from a single clone for each group. For scatter plots, data were mean ± SEM, *n* = 9 combined from three CB-ECFC clones; *** *p* < 0.001, **** *p* < 0.0001 versus normoxia, unpaired Student’s *t*-test.

To investigate impact of acute hypoxia on CB-ECFC angiogenic functions, cells were exposed to 1% O_2_ for 48 h and compared to normoxia (21% O_2_). CB-ECFCs showed reduced tube formation ability after incubation in 1% O_2_, indicated by decreased tube area by tubulogenesis assay (Figure 1A). This was observed together with clear induction of hypoxia-inducible factor-1α (HIF-1α) as confirmation that the cells were under hypoxic stress (Figure 1B) and exhibited reduced metabolic activity (Figure 1C). In addition, generation of both superoxide and hydrogen peroxide, which were established as key regulators of ECFCs and endothelial cell function, were markedly reduced in CB-ECFCs exposed to hypoxia versus normoxia controls (Figure 1D,E).

### 3.2. CB-ECFC NOX4 Signalling Is Suppressed by Hypoxia

**Figure 2:**
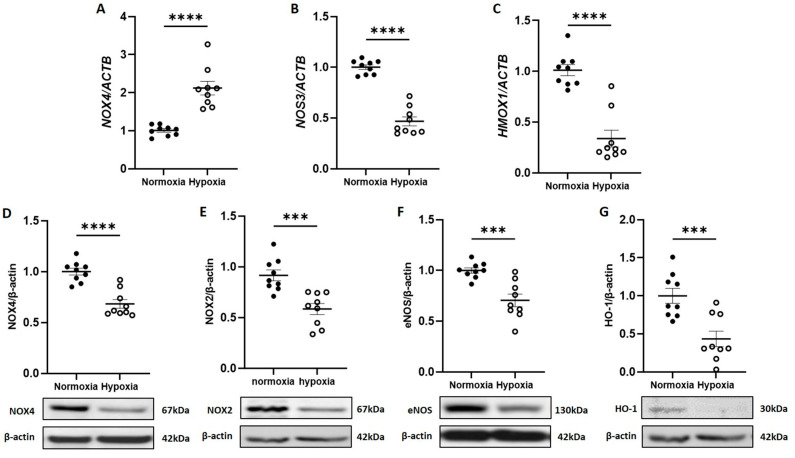
**CB-ECFC NOX4 activity and signalling was reduced by hypoxia.** CB-ECFCs were cultured under hypoxia or normoxia for 48 h prior to the analysis of (**A**–**C**) mRNA expression of *NOX4*, *NOS3* and *HMOX-1* and (**D**–**G**) protein expression of NOX4, NOX2, eNOS and HO-1 using qRT-PCR and Western blot, respectively, with normalisation to *ACTB*/β-actin as reference control. Representative blots are shown from a single clone for each group. For scatter plots, data were mean ± SEM; *n* = 9 combined from three CB-ECFC clones; *** *p* < 0.001, **** *p* < 0.0001 versus normoxia, unpaired Student’s *t*-test.

As our previous work identified NOX4 NADPH oxidase as a major positive regulator of CB-ECFC angiogenic signalling, subsequent studies aimed to interrogate its specific impact in the setting of experimental hypoxia, which is associated with reduced CB-ECFC function and ROS generation. Whilst qRT-PCR analysis indicated that *NOX4* mRNA expression was induced in CB-ECFCs subjected to 48 h hypoxia (Figure 2A), NOX4 protein levels decreased as an indicator of suppressed constitutive activity (Figure 2D).

Consistent with these data, both mRNA and protein expressions of endothelial nitric oxide synthase (*NOS3* or eNOS; Figure 2B,F) and heme oxygenase (*HMOX1* or HO-1; Figure 2C,G), as established redox-sensitive downstream targets of NOX4, which were dysregulated in response to hypoxia in an HIF-1α-dependent manner [19] and were decreased in CB-ECFCs exposed to hypoxia versus normoxia. Although these results suggested that hypoxia could differentially regulate *NOX4* mRNA expression and protein translation in CB-ECFCs, they clearly indicated reduction in downstream redox-sensitive signalling which was in line with reduced angiogenic function and ROS production. Consistent with our previous study [16], *NOX2* mRNA expression could not be detected in CB-ECFCs cultured under normoxia and was not induced by hypoxia, although NOX2 protein expression was reduced (Figure 2E).

### 3.3. NOX4 Overexpression Partially Restores CB-ECFC Angiogenic Function in Hypoxia via Activation of Nrf2

**Figure 3:**
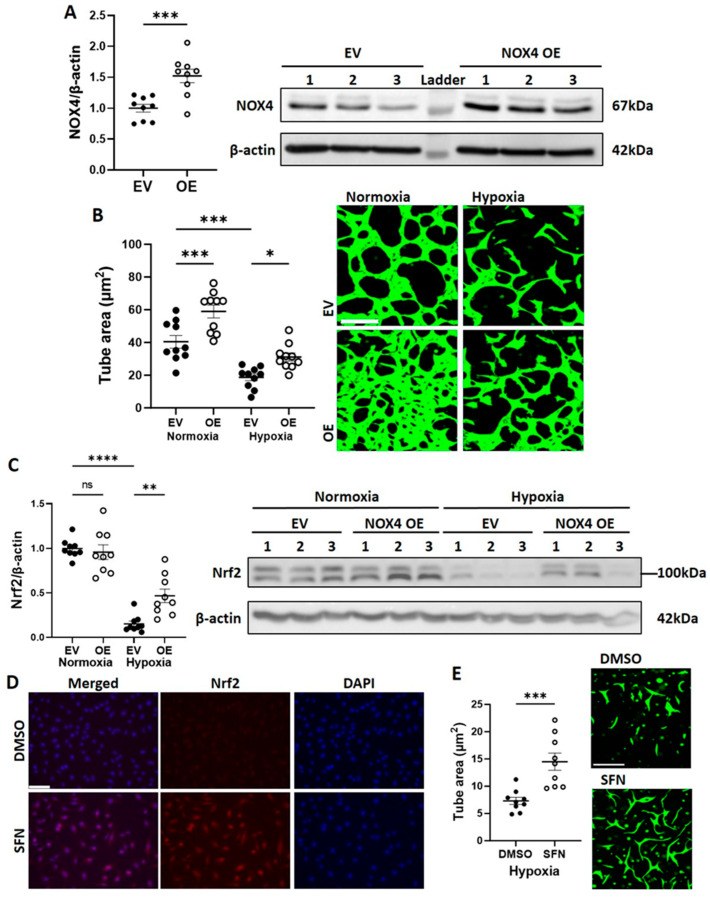
**NOX4 overexpression partially restored CB-ECFC angiogenic function in hypoxia via activation of Nrf2.** CB-ECFCs were electroporated with either an EV or NOX4 OE plasmid for 48 h and (**A**) protein was collected at 72 h for confirmation of NOX4 OE or incubated under hypoxia or normoxia for a further 48 h prior to quantification of (**B**) tube area using Matrigel tubulogenesis assay or (**C**) Nrf2 protein expression using Western blot with normalisation to β-actin as reference control. CB-ECFCs were treated with sulforaphane (SFN; 4 µmol/L for 4 h) prior to (**D**) visualisation of Nrf2 activation by immunocytochemistry, and (**E**) quantification of tube area using Matrigel tubulogenesis assay after further incubation under hypoxia for 48 h. Representative blots are shown from a single clone for each group. For scatter plots, data were mean ± SEM; *n* = 9 combined from three CB-ECFC clones; * *p* < 0.05, ** *p* < 0.01, *** *p* < 0.001, **** *p* < 0.0001 versus the relevant EV/DMSO control, unpaired Student’s *t*-test or one-way ANOVA with Bonferroni post-hoc testing.

Further to the finding that NOX4 protein expression and signalling were suppressed in CB-ECFCs exposed to hypoxia, we next investigated whether NOX4 OE, which we previously showed to promote basal angiogenic function [16], could rescue hypoxia-induced CB-ECFC dysfunction. Cells were transfected with either an EV or NOX4 OE plasmid by electroporation, which resulted in an ~1.5-fold induction of NOX4 protein expression (Figure 3A) prior to culture in hypoxia or normoxia for 48 h. Reduced tube formation in EV-transfected CB-ECFCs exposed to hypoxia was partially restored by NOX4 OE, which also improved angiogenic function in cells subjected to normoxia (Figure 3B). These data were consistent with our previous report, while also highlighting the importance of NOX4 signalling for supporting CB-ECFC angiogenic function under hypoxia. The observed pro-angiogenic effects of NOX4 appeared to be at least partly dependent on its established downstream target, Nrf2. These effects were elevated in NOX4 OE versus EV-transfected CB-ECFCs exposed to hypoxia (Figure 3C), while Nrf2 activation with sulforaphane rescued hypoxia-induced angiogenic dysfunction (Figure 3D,E).

### 3.4. CB-ECFC Gene Expression Is Differentially Regulated in Hypoxia

**Figure 4:**
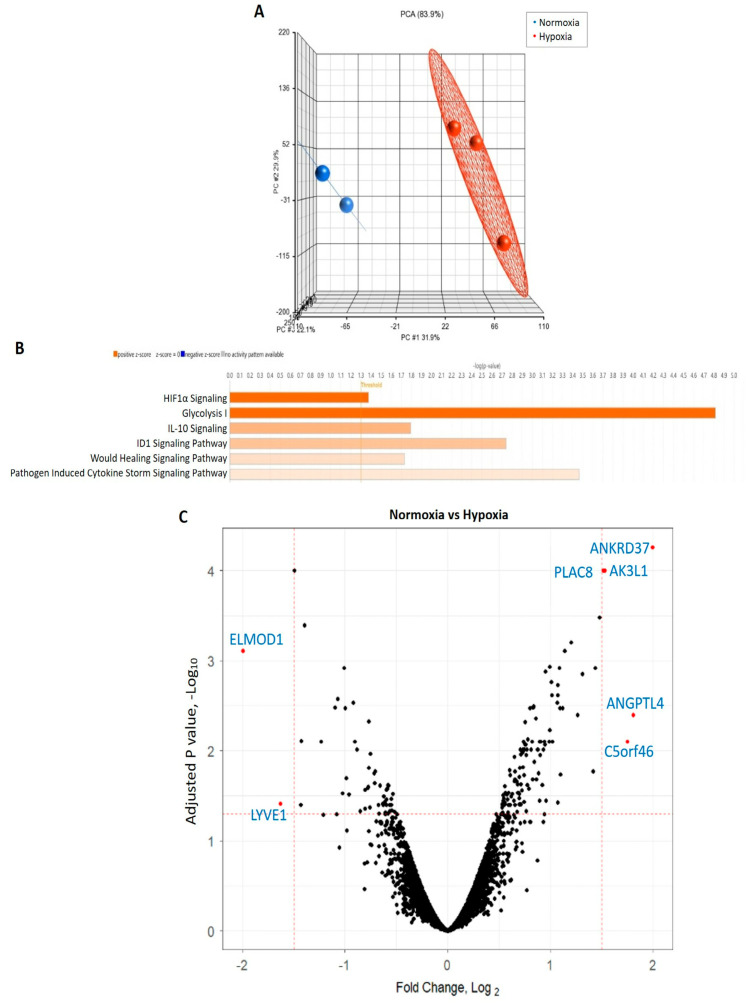
**CB-ECFC gene expression was differentially regulated in hypoxia.** (**A**) Principal component analysis generated using Partek showing distinct clustering of gene expression profiles in CB-ECFCs exposed to hypoxia (*n* = 3) or normoxia (*n* = 2) for 48 h. (**B**) Applying our thresholds (fold change + 1.5, *p* < 0.05) to IPA identified six activated pathways (z-score > 2), which are shown in orange; threshold -log *p* > 1.3. (**C**) Volcano plot generated from R package showing DEGs between treatment conditions. Each dot indicates a single gene; black represents no difference, red represents statistically significant log fold change ± 1.5; adjusted *p* value < 0.05.

Microarray analysis of CB-ECFCs exposed to either normoxia or hypoxia for 48 h was performed in order to investigate cell transcriptional responses in relation to NOX4 signalling. Principal component analysis highlighted distinct separation of gene expression profiles based on treatment conditions (Figure 4A). A gene list generated from Partek was imported to IPA by applying our fold change of ±1.5 and *p* < 0.05 thresholds. Applying a z-score > 2 as the threshold for significant activation, six canonical pathways were found to be significantly activated, which were ranked according to their activity (Figure 4B). Detailed interrogation of differential expression between CB-ECFCs exposed to hypoxia versus normoxia indicated several highly dysregulated genes with a fold change greater than ± 1.5 and −logP > 1.3, as shown in the volcano plot (Figure 4C). Seven differentially expressed genes (DEGs) were identified using the limma package within the R statistical environment, which were either significantly upregulated (*ANKRD37*, *AK3L1*, *PLAC8*, *ANGPTL14*, *C5orf46*) or downregulated (*ELMOD1*, *LYVE1*) in hypoxia versus normoxia-treated CB-ECFCs. Notably, the most activated pathways in CB-ECFCs exposed to hypoxia were found to be related to HIF-1α signalling and glycolysis, which was consistent with our earlier observations (Figure 1B,C).

### 3.5. PLAC8 Is a Key Upstream Negative Regulator of CB-ECFC NOX4 Signalling in Hypoxia

**Figure 5:**
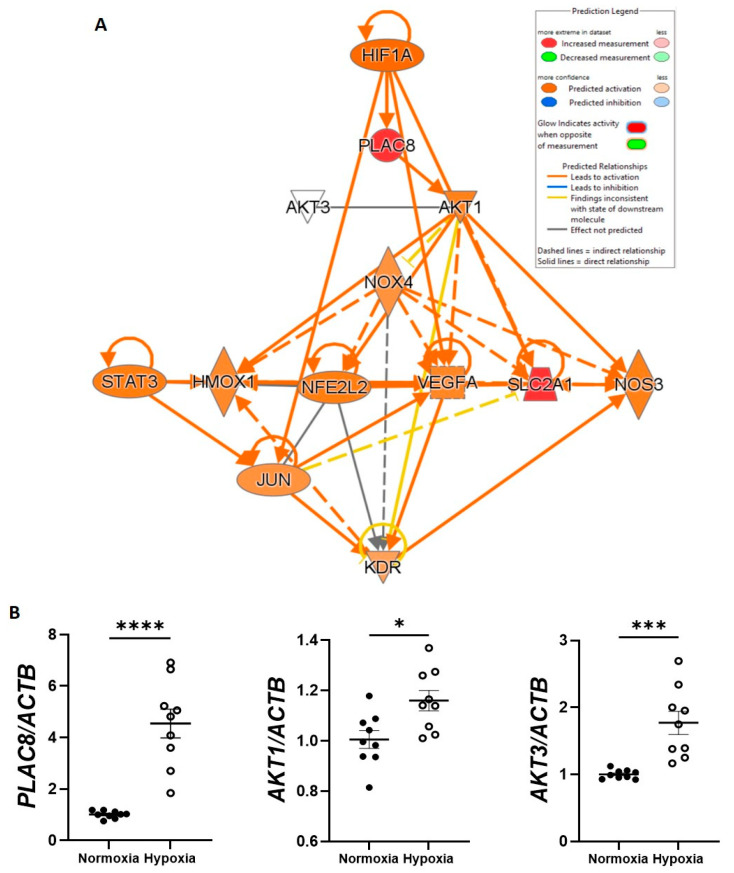
**PLAC8 was a key upstream negative regulator of CB-ECFC NOX4 signalling in hypoxia.** (**A**) IPA network analysis of CB-ECFCs exposed to hypoxia or normoxia for 48 h. The node colour represents upregulated (red) and downregulated (green) genes, while orange and blue colouring represents genes with predicted activation and inhibition, respectively. (**B**) mRNA expression of *PLAC8*, *AKT1* and *AKT3* using qRT-PCR with normalisation to *ACTB* as the reference control. For scatter plots, data were mean ± SEM; *n* = 9 combined from three CB-ECFC clones; * *p* < 0.05, *** *p* < 0.001, **** *p* < 0.0001 versus normoxia, unpaired Student’s *t*-test.

Having established strong influence of hypoxia on CB-ECFC HIF-1α-mediated gene expression, detailed network analysis was performed using IPA to interrogate the specific interaction between DEGs and NOX4 signalling and their downstream functions (Figure 5A). Most notably, of the seven DEGs in hypoxia versus normoxia-treated CB-ECFCs identified by R analysis (Figure 4C), upregulation of *PLAC8* expression in the dataset was predicted to be associated with upstream stabilisation of *HIF-1α* and was highlighted as an inhibitor of downstream *NOX4* signalling through interaction with *AKT1* and *AKT3*. Furthermore, PLAC8/NOX4 signalling in this setting was linked to regulation of key eNOS-dependent cellular functions, including VEGFR2 (*KDR*)-dependent angiogenesis and extracellular matrix remodelling, as established CB-ECFC effector processes, while *NFE2L2* (Nrf2), *STAT3* and *JUN* were identified as intermediate transcription regulators. 

Increased CB-ECFC mRNA expression of *PLAC8*, *AKT1* and *AKT3*, as potential upstream regulators of NOX4 in hypoxia (identified through IPA network analysis), was validated using qRT-PCR (Figure 5), while enhanced protein activation of VEGFR2, STAT3 and c-Jun was later confirmed using phospho-kinase proteome array and/or Western blot in CB-ECFCs subjected to PLAC8 KD and exposure to hypoxia, further supporting these genes/proteins as key mediators of HIF-1α-dependent signaling.

### 3.6. PLAC8 Is a Key Upstream Negative Regulator of CB-ECFC NOX4 Signalling in Hypoxia

**Figure 6:**
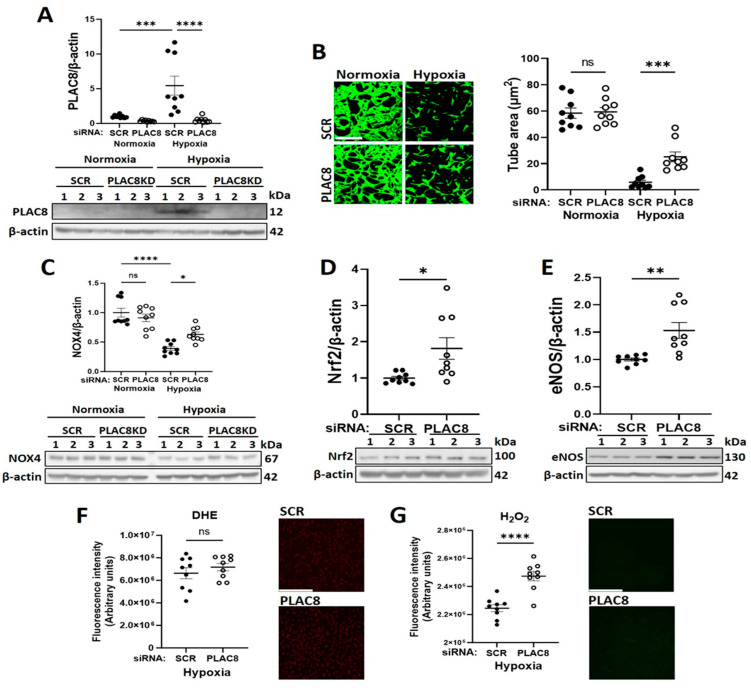
**Inhibition of PLAC8 expression enhanced CB-ECFC NOX4-mediated angiogenic signalling in hypoxia.** CB-ECFCs were transfected with either PLAC8-targeting siRNA or non-targeting SCR control for 24 h prior to culture in hypoxia or normoxia for 48 h. (**A**) Confirmation of PLAC8 KD by Western blot with normalisation to β-actin as reference control. (**B**) Tube area confirmed by Matrigel tubulogenesis assay. (**C**–**E**) Protein expression of NOX4, Nrf2 and eNOS by Western blot with normalisation to β-actin as reference control. Production of (**F**) superoxide confirmed using DHE staining and (**G**) hydrogen peroxide using a commercial assay with Image-J quantification. For scatter plots, data were mean ± SEM; *n* = 9 combined from three CB-ECFC clones; * *p* < 0.05, ** *p* < 0.01, *** *p* < 0.001, **** *p* < 0.0001 versus relevant SCR control, unpaired Student’s *t*-test or one-way ANOVA with Bonferroni post-hoc testing.

**Figure 7:**
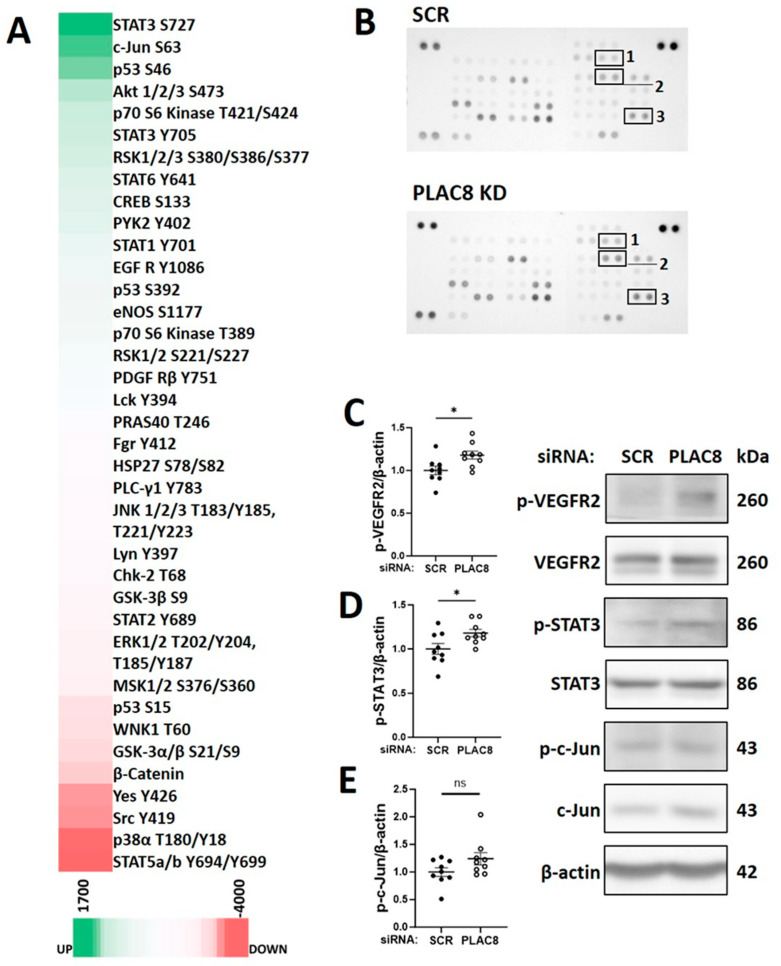
**PLAC8 knockdown promoted increased phosphorylation of VEGFR2, STAT3 and c-Jun as downstream mediators of CB-ECFC angiogenic signalling in hypoxia.** CB-ECFCs were transfected using either PLAC8-targeting siRNA or non-targeting SCR control for 24 h prior to culture in hypoxia or normoxia for 48 h and protein extraction. (**A**) A heatmap of relative changes in protein kinase phosphorylation using a human phospho-kinase proteome profiler array. The green and red colouring represent increased and decreased expressions, respectively. (**B**) Original blots incubated with 300µg of pooled protein lysate of triplicates from three CB-ECFC clones (1: c-Jun; 2: p53; 3: STAT3). (**C**–**E**) Protein expression of p-VEGFR2, p-STAT3 and p-c-Jun using Western blot with normalisation to β-actin as reference control. For scatter plots, data were mean ± SEM; *n* = 9 combined from three CB-ECFC clones; * *p* < 0.05 versus SCR control, unpaired Student’s *t*-test.

To further clarify implication of PLAC8 as an upstream inhibitor of NOX4 signalling in CB-ECFCs exposed to hypoxia, its specific regulatory role in this setting was interrogated in a series of mechanistic experiments employing targeted siRNA KD. Cells were transfected with either siRNA against PLAC8 or SCR control for 24 h prior to culture in hypoxia or normoxia for 48 h. PLAC8 protein was found to be expressed at low levels in CB-ECFCs exposed to normoxia but was markedly induced (~ six-fold) by hypoxia, whilst siRNA transfection resulted in almost complete KD of PLAC8 expression in both normoxia and hypoxia-treated cells (Figure 6A). Consistent with our bioinformatics network analysis, tube formation assessed using 3D Matrigel assay was improved in PLAC8 KD versus SCR control CB-ECFCs after hypoxia exposure, while angiogenic function remained similar in CB-ECFCs subjected to the same transfection protocol and maintained under normoxia (Figure 6B). Furthermore, protein expression of NOX4 was increased in PLAC8 KD versus SCR control CB-ECFCs under hypoxia (but not normoxia), as well as Nrf2 and eNOS as established downstream targets, confirming PLAC8 as a key inhibitor of NOX4 and downstream pro-angiogenic signalling (Figure 6C–E). Interestingly, although our previous study highlighted NOX4-derived superoxide as a positive regulator of CB-ECFC function in normoxia [16], superoxide generation was not impacted by PLAC8 KD in hypoxia (Figure 6F), while production of hydrogen peroxide was increased (Figure 6G), indicating divergent NOX4-mediated ROS signalling between culture conditions. Further mechanistic analysis based on a human phospho-kinase proteome profiler array of protein extracted from PLAC8 KD versus SCR control CB-ECFCs after 48 h of hypoxia exposure highlighted differential regulation of several key angiogenesis-related transcription factors, which were also identified in our previous network analysis (Figure 4A) and were highly likely to mediate downstream signalling in this setting (Figure 7A,B). Specifically, activation of NOX4 signalling secondary to inhibition of PLAC8 expression resulted in marked upregulation of signal transducer and activator of transcription 3 (STAT3), transcription factor Jun (c-Jun), and cellular tumour antigen p53 as candidate downstream promotors of redox-sensitive CB-ECFC VEGF-dependent angiogenic functions in this setting. Their phosphorylation was confirmed by Western blot (Figure 7C–E), further supporting these genes/proteins as key mediators of HIF-1α-dependent signaling.

## 4. Discussion

**Figure 8:**
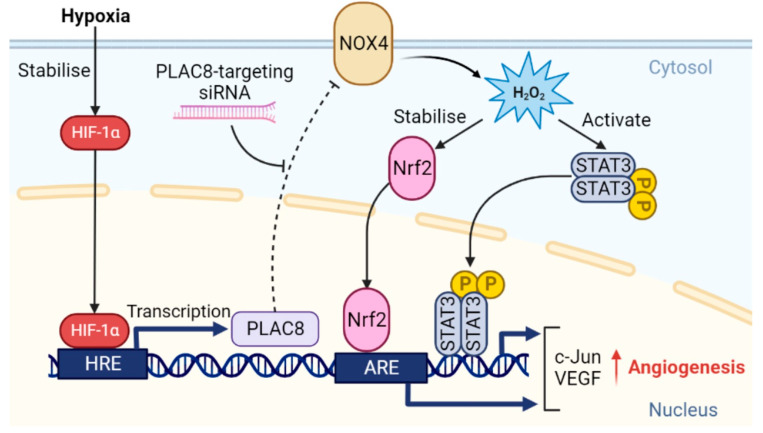
**PLAC8 regulated NOX4-dependent pro-angiogenic signalling in CB-ECFCs exposed to experimental hypoxia.** Summary schematic indicating that stabilisation and translocation of HIF-1α to HRE of the CB-ECFC nucleus under hypoxic conditions led to *PLAC8* transcription and downregulation of NOX4 expression (dotted arrow). In the presence of PLAC8-targeting siRNA, NOX4 expression was restored, resulting in generation of hydrogen peroxide, downstream stabilisation of Nrf2 and induction of angiogenesis via activation of STAT3-c-Jun-VEGF signalling. ARE: antioxidant response element; HRE: hypoxia response element.

Ischaemic cardiovascular disease is a leading global cause of morbidity and mortality, which is largely determined by impaired revascularisation and characterised by tissue hypoxia and endothelial dysfunction as major pathological drivers [1,2]. It is well established that CB-ECFCs demonstrate enhanced angiogenic capability compared to mature endothelial cells, underlying their evident therapeutic potential. However, CB-ECFC angiogenic function becomes suppressed upon introduction to ischaemic tissue due to imbalance between metabolic supply and demand and local hypoxia, which significantly reduces efficacy and represents a major barrier to clinical translation [20]. In this regard, our current study presented convincing data, indicating that reactivation of PLAC8–NOX4 signalling partially restored CB-ECFC function in experimental hypoxia via induction of downstream pro-angiogenic pathways. As summarised in Figure 8, we showed that siRNA KD of PLAC8 enhanced NOX4 expression and hydrogen peroxide generation in hypoxic CB-ECFCs, leading to stabilisation of Nrf2, activation of STAT3 and downstream induction of cJun-VEGF-mediated angiogenic signalling, which was reflected by enhanced tube formation. These intriguing findings implicated this novel signalling axis as a potential target for increasing resilience of therapeutic CB-ECFCs within the ischaemic cardiovascular disease microenvironment by providing protection against hypoxia-induced dysfunction.

We initially demonstrated that CB-ECFC angiogenic function and metabolic activity were suppressed in experimental hypoxia in a HIF-1α-dependent manner (Figure 1A,B), as confirmed in previous reports [21] and consistent with the known function of HIFs as principal regulators of metabolic and angiogenic through activation of various hypoxia response elements (HREs) [22]. In this regard, it was not surprising that the two most activated pathways identified through microarray analysis of CB-ECFCs exposed to hypoxia versus normoxia were HIF-1α signalling and glycolysis, which are critical for cell survival and ATP production under hypoxic conditions (Figure 4B). We also highlighted activation of ID1 signalling in hypoxic CB-ECFCs, which was established as an important promoter of NFkB-dependent endothelial progenitor cell proliferation [23], while activation of IL10 signalling could indicate an anti-inflammatory response. The latter was supported by a report that TNF-α-induced IL10 expression improved tube formation and migration in endothelial progenitor cells via upregulation of STAT3 [24], which we also highlighted as a downstream promotor of PLAC8–NOX4 signalling in CB-ECFCs. However, it should be noted that CB-ECFC proliferation and angiogenic function were suppressed by hypoxia in our study (Figure 1C), suggesting that likely adaptive induction of ID1 and IL10 signalling was unable to impact cell function under these conditions.

Imbalanced energy production in hypoxia led to interruption of protein synthesis in order to reduce energy consumption as an adaptive response to cellular stress [25]. In this regard, we found that hypoxia-induced CB-ECFC dysfunction was associated with significant downregulation of NOX4 and downstream eNOS and HO-1 expression (Figure 2D,F,G), which we previously reported as a positive regulator of CB-ECFC angiogenic function under normoxic conditions that promoted post-ischaemic revascularisation [16]. It is interesting to note that while NOX4 protein expression was reduced with hypoxia in parallel with decreased generation of both superoxide and hydrogen peroxide (Figure 1D,E), *NOX4* mRNA expression was increased (Figure 2A). It was likely that this represented an ineffective compensatory response to protect CB-ECFC function through induction of NOX4-mediated ROS signalling, which promotes endothelial cell angiogenic capacity [26]. Indeed, while hypoxia promotes oxidative stress in endothelial cells, largely secondary to mitochondrial superoxide generation and dysfunctional eNOS signalling [27,28], CB-ECFCs appear to show an opposite response, aligning with our conclusion that ROS are protective in this setting. Hypoxia is also known to negatively impact expression and activity of eNOS in endothelial cells, including coronary artery, umbilical vein and pulmonary artery [29,30,31], which was consistent with our observation of reduced eNOS expression and signalling in hypoxic CB-ECFCs. This aligned with reduced expression of HO-1, an established regulator of ROS-dependent pro-angiogenic signalling, which was suppressed in hypoxia via upregulation of the transcription factor BACH1 in response to HIF-1α induction [32,33]. Taken together, these initial data were supportive of a critical role for NOX4 in determining hypoxia-induced CB-ECFC angiogenic dysfunction through regulation of ROS production and redox homeostasis. It was interesting that NOX2 protein expression was also reduced in hypoxic CB-ECFCs (Figure 2E), highlighting a potential role for this NOX isoform, while noting that NOX4 was likely to be more significant in this context based on our previous observation that it was the most highly expressed NOX isoform in healthy CB-ECFCs and a major regulator of angiogenic function [16].

As confirmation of our previous work, we found that induction of NOX4 signalling in CB-ECFCs by plasmid OE increased angiogenic capacity in normoxic conditions [16]. The present study showed this to also partially restore angiogenic dysfunction in CB-ECFCs exposed to hypoxia (Figure 3B). Furthermore, our new data indicated that pro-angiogenic effects of NOX4 signalling in this setting were mediated via stabilisation of Nrf2 as an established downstream target (Figure 3C–E). Indeed, Nrf2 KD was reported to inhibit endothelial cell migration and angiogenesis in hypoxia [34], functions which are promoted in endothelial progenitor cells by induction of NOX4-derived hydrogen peroxide and associated modulation of inflammation [35]. However, perhaps the most novel finding of the current study was identification of PLAC8 as a negative regulator of NOX4-mediated angiogenic signalling in hypoxic CB-ECFCs. PLAC8 is a member of the cornifelin family, which was first discovered through microarray analysis of mouse placenta and embryo and regulates AKT1 activation via transient binding to the C/EBPβ promoter [36,37,38]. Of relevance to the current study, PLAC8 is highly expressed in stem cells during embryonic development [39], while suppression of PLAC8 expression promotes proliferation and regenerative capacity of pluripotent stem cells via induction of JNK-dependent signalling [40]. There are emerging reports linking PLAC8 to disease progression, including cancer [41,42] and diabetes [36], although specific mechanisms are poorly defined. For example, one study found PLAC8 induction to promote cell proliferation in breast cancer [42], while another reported inhibitory actions in hepatocellular carcinoma [43]. However, there is a paucity of understanding of the role of PLAC8 expression in endothelial progenitor cells, which appears to be limited to reported upregulation in ECFCs isolated from neonates exposed to gestational diabetes, inhibition of which led to an improved proliferation and senescence [36]. It is interesting to note that PLAC8 expression was detectable at very low levels in CB-ECFCs cultured in normoxia, while PLAC8 KD did not impact tube formation under the same conditions (Figure 6A,B), suggesting that PLAC8 signalling may not be physiologically significant in this context. However, PLAC8 expression was markedly induced in CB-ECFCs exposed to hypoxia together with *AKT1* and *AKT3* as established targets (Figure 5), which occurred in parallel with reduced tube formation. However, PLAC8 KD under these conditions resulted in partial restoration of angiogenic capacity and reactivation of NOX4-mediated signalling, including downstream Nrf2 and eNOS (Figure 6A–E). Therefore, while PLAC8 appeared to be of limited functional relevance in CB-ECFCs under physiological conditions, these new data indicated a likely important pathological role for PLAC8 induction under hypoxic conditions as a potential target for promoting CB-ECFC angiogenic function within the hostile disease microenvironment.

We previously reported that NOX4 promoted CB-ECFC angiogenic function in normoxia via specific induction of superoxide production [16]. However, while levels of both superoxide and hydrogen peroxide were reduced in CB-ECFCs exposed to hypoxia, PLAC8 KD only restored hydrogen peroxide generation but did not impact superoxide (Figure 6F,G), suggesting that NOX4 angiogenic signalling in hypoxia was determined by different ROS. Although this may be surprising, it was important to consider the established complexity of NOX signalling in endothelial cells, with critical factors such as the ROS type, concentration and subcellular localisation defining the downstream actions [44]. For example, hydrogen peroxide may function as a second messenger at certain intracellular threshold levels, suggested to be in the range of 10–100 µmol/L, but may be detrimental at higher concentrations [45]. NOX4 may also generate highly diffusible hydrogen peroxide directly or secondary to dismutation of superoxide [46] as a potential mechanism underlying apparently divergent ROS signalling in normoxic and hypoxic CB-ECFCs. Nonetheless, observed promotion of NOX4 expression and hydrogen peroxide production secondary to PLAC8 KD in hypoxic CB-ECFCs was linked to induction of key transcriptional activators associated with VEGF-dependent angiogenesis, including STAT3 and c-Jun (Figure 5A and Figure 7A–E), which were also regulated by Nrf2-mediated ROS signalling [47,48,49] and consistent with our previous report that miR-130a enhanced ECFC vasoregenerative capacity via activation of VEGFR2/STAT/3/HIF-1α signalling [50]. In addition, PLAC8 KD was found to activate p53, a critical stress response protein reported to stabilise Nrf2 under pathological stresses, such as hypoxia [51]. Taken together, these data defined a clear mechanism by which PLAC8 expression regulated NOX4-dependent hydrogen peroxide and downstream STAT3-c-Jun-VEGF-mediated angiogenic signalling (Figure 5A and Figure 8).

## 5. Conclusions

In summary, the findings of this study clearly implicated PLAC8-NOX4 signalling axis as an important regulator of CB-ECFC angiogenic function in hypoxia, while defining detailed downstream pathways that may be clinically significant. While CB-ECFCs hold evident potential for treatment of ischaemic cardiovascular disease, a major barrier to translation is reduced angiogenic function upon introduction to hostile tissue microenvironments, which are often characterised by hypoxia. In this regard, it is plausible that therapeutic activation of NOX4 signalling through targeted inhibition of PLAC8 could represent a potential novel approach for protecting CB-ECFCs from such disease stresses, thereby enhancing efficacy for promotion of vascular regeneration and repair.

## Data Availability

The original data are available from the corresponding authors upon request.
